# Report of the Fifth Post-Kala-Azar Dermal Leishmaniasis Consortium Meeting, Colombo, Sri Lanka, 14–16 May 2018

**DOI:** 10.1186/s13071-020-04011-7

**Published:** 2020-03-30

**Authors:** Eduard E. Zijlstra, Amresh Kumar, Abhijit Sharma, Suman Rijal, Dinesh Mondal, Satyabrata Routray

**Affiliations:** 1 Drugs for Neglected Diseases Initiative, 15 Chemin Louis Dunant, 1202 Geneva, Switzerland; 2 PATH, 15th Floor, Dr. Gopaldas Building, 28 Barakhamba Road, Connaught Place, New Delhi, 110001 India; 3 Drugs for Neglected Diseases Initiative, PHD House, 3rd Floor, 4/2 Siri Institutional Area, New Delhi, 110016 India; 4 Nutrition and Clinical Services Division, International Center For Diarrheal Disease Research, Bangladesh (icddr,b), 63 Shaheed Taj Uddin Ahmed Sarani, Mohakhali, Dhaka, Bangladesh

**Keywords:** Post-kala-azar dermal leishmaniasis, Epidemiology, Diagnosis, Parasitology, Immunology, Treatment, Recommendations

## Abstract

The 5th Post-Kala-Azar Dermal Leishmaniasis (PKDL) Consortium meeting brought together PKDL experts from all endemic areas to review and discuss existing and new data on PKDL. This report summarizes the presentations and discussions and provides the overall conclusions and recommendations.

## Background

The Post-Kala-azar Dermal Leishmaniasis (PKDL) Consortium was founded in 2012 by the Drugs for Neglected Diseases *initiative* (DND*i*), Geneva, Switzerland, and PATH, New Delhi, India, to promote research, advocacy, training and communication in the field of PKDL as it relates to visceral leishmaniasis (VL), also known as kala-azar (KL). At the founding meeting in Delhi, India, a mission statement was formulated: “The PKDL Consortium is committed to promoting and facilitating activities that lead to better understanding of all aspects of PKDL that are needed for improved clinical management and to achieve control of PKDL and VL” [1].

The Consortium acts as a forum for all those interested in PKDL to be in contact with each other and meet in person every one to two years to review research outcomes, translate these into policy, and identify current needs. This is particularly relevant for bringing together scientists from Asia and Africa, the two main endemic regions for VL, where PKDL is common but where the epidemiology, clinical presentation, and perspectives for control are different. In the Indian subcontinent (ISC), the Kala-azar Elimination Programme (KAEP) is proving successful in reducing VL incidence and is moving towards the consolidation phase, whereas PKDL needs to be urgently addressed in Africa to interrupt the ongoing outbreak and to prevent a future upsurge.

This report summarizes the presentations and discussion at the 5th meeting of the Consortium in Colombo, Sri Lanka, 14–16 May 2018. The agenda of the meeting and the list of participants are provided in Additional files 1 and 2.

## Session: Epidemiology, disease burden, and clinical presentation

### Epidemiology of PKDL in Asia and Africa (Presenter: Eduard E. Zijlstra)

#### Definition and background

PKDL is an intermediate disease state between VL and cure of VL. The clinical manifestations are driven by the immune response to the parasite. In VL, there is a predominantly T-helper (Th) 2 response characterized by high levels of interleukin-10 (IL-10), while in cured VL the immune response is predominantly Th1 with high levels of interferon-gamma (IFN-γ). In PKDL, the immune response has characteristics of both Th2 and Th1, and it has been suggested that there is a dissociation between the immune response in the skin (Th2), possibly under the influence of ultraviolet light, and the systemic immune response (Th1) as the result of successful treatment of VL [2]. In Asia, PKDL follows VL with an interval of 0 to 3 years, and the PKDL peak follows that of VL with an offset of 2 years [3]. In Africa (Sudan) the interval is 0–12 months; there are anecdotal reports suggesting that sporadic PKDL cases may trigger VL outbreaks [4].

#### Disease burden

**Fig. 1:**
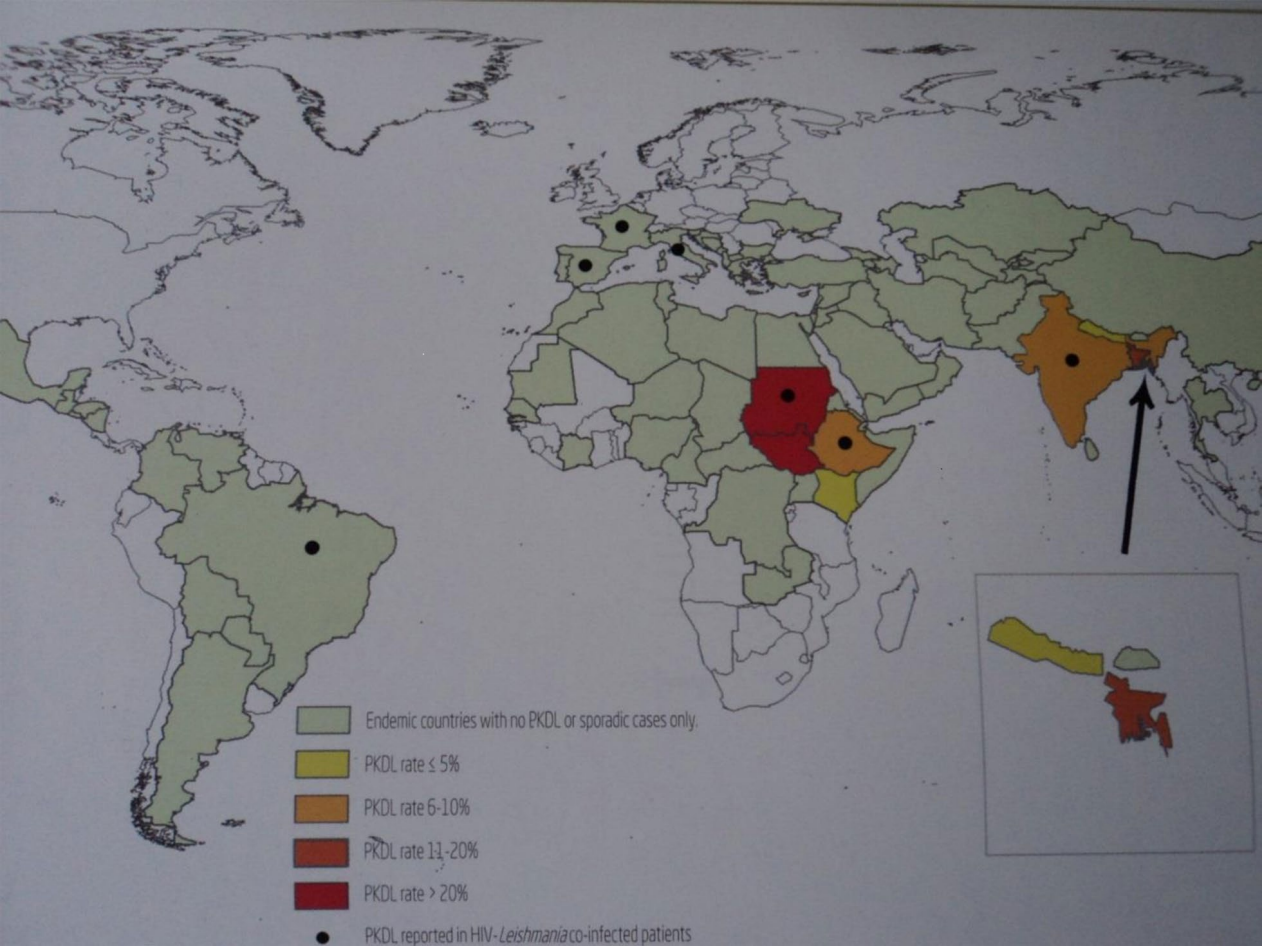
Worldwide distribution of PKDL. *Source*: Reproduced with permission from WHO. Zijlstra EE, Alvar J, editors. (2012) The post kala-azar dermal leishmaniasis (PKDL) atlas. A manual for health workers. Geneva: World Health Organization [54]

The disease burden is not known. High PKDL-endemic areas are shown in Fig. 1. The highest point prevalence in a field study in Africa was 4.8/100, while it was 4.8/1000 in Asia [5].The PKDL rate (i.e. the proportion of cases where PKDL follows VL) is less than 5% in Nepal, 10% in India and 20% in Bangladesh, while it is up to 60% in Africa (Sudan) and 20% in Ethiopia.[5] HIV is a risk factor for more frequent and more severe disease [6]. In Asia, PKDL is commonly macular, while in Africa the papulonodular form is more common. Self-healing is common in Sudan (85% in 12 months), while it is assumed to be rare in Asia where all patients are treated.

#### Risk factors

PKDL predominantly occurs in *L. donovani*-endemic areas, and is rare in *L. infantum* infection. PKDL has been described after VL treatment with all currently available drugs. Incomplete treatment, or treatment with substandard drugs or low doses, seems to be an important risk factor. In both Africa and Asia, young age is a risk factor; in Africa, VL and PKDL is more common among very young children, while in Asia it is more common among adolescents. High IL-10 levels in the skin and high C-reactive protein levels in the blood during VL are risk factors for PKDL [7, 8]. Incomplete clearance of parasites also plays a role; persistence of parasites as demonstrated by polymerase chain reaction (PCR) in a lymph node aspirate after VL treatment leads to higher PKDL rates [9]. PKDL is more common and more severe in people coinfected with HIV [6].

#### Role of PKDL in transmission

In both Asia and Africa, it is thought that PKDL plays a role in transmission in the interepidemic episodes, as well as during outbreaks. No consensus exists on the need for treatment of all PKDL patients in Africa, and only those with severe disease are treated. This should be seen in the context of the ongoing 25-year VL outbreak. In Asia, all patients are treated with miltefosine (MF), despite the (gastrointestinal) side effects, its teratogenic potential, and long duration of treatment (3 months). The number of VL cases has dropped considerably, and active case detection (ACD) and treatment of PKDL may have been important factors. More information is needed on the infectivity of PKDL and other manifestations such as active VL, ex-VL and HIV-VL, as well as on the optimal method for case detection; there is also a need for safer effective drugs. In addition, it is not known how low the PKDL rate needs to be to prevent new outbreaks.

### Cohort observational study to estimate the cumulative incidence of PKDL in VL patients treated with three regimens in Bihar (Presenter: Suman Rijal)

#### Overview

In India (Bihar), a cohort of 1622 KA patients treated between 2012 and 2015 were followed up in 2016 and 2017 to determine the occurrence of PKDL. The VL patients had been treated with one of the 3 following regimens: (i) combination treatment with miltefosine and paromomycin (MF+PM); (ii) combination treatment with AmBisome^®^ (AMB, Gilead Sciences, Inc., Foster City CA, USA) and MF (AMB+MF); and (iii) single-dose AmBisome (SDA) treatment.

The cure rates of these regimens for VL were 91.4%, 88.8% and 96.9%, respectively[10]. During active follow-up, the occurrence of PKDL, interval after VL, age, gender, and clinical presentation were recorded. An algorithm was used for diagnosis of probable PKDL (presence of skin lesion with positive rK39 test) and confirmed PKDL (slit skin smear or quantitative polymerase chain reaction (qPCR)-positive for *L. donovani*). The cumulative incidence of PKDL was 6.3%, with a PDKL rate of 4.8%, 5.7% and 9.2% for the SDA, AMB+MF, and MF+PM regimens, respectively.

The mean age of PKDL patients was younger than those without PKDL (21 *vs* 28 years, respectively); 53% of PKDL cases were females, and 38% of those with no PKDL were female. Macular lesions were most common in all regimens (84–100%). The onset in the AMB+MF regimen was delayed compared to the other regimens (30 months *vs* 25 for SDA and 24 months for MF+PM).

PKDL developed in 6.3% of VL patients after at least 24 months after VL treatment, with higher cumulative incidence in those treated with MF+PM. Additional analysis is needed to adjust for variable time of follow-up. PKDL was more common in young and female patients.

#### Discussion

The PKDL rates included probable and confirmed PKDL, and those who were parasite-positive were confirmed by PCR. PM and MF are losing their effect in India, which might explain the high PKDL rates. Although the parasites isolated from PKDL patients in one study showed lower sensitivity to MF, no MF-resistant natural strains have been isolated so far. Access to MF and PM should remain restricted to the public sector to prevent the development of resistance.

### Post kala-azar dermal leishmaniasis in Nepal (Presenters: Bibek Lal and Surendra Uranw)

#### Overview

In a study from 2010, 680 ex-VL cases were screened for PKDL; of these 82% were treated for VL with sodium stibogluconate (SSG), 10% with amphotericin B, and 8% with MF. Of the PKDL cases, 5.4% (*n* = 37) were clinically suspected and 2.4% (*n* = 16) were confirmed by slit skin smear. PKDL occurred on average 23 months after treatment; the PKDL rate was 1.4%, 2.5% and 3.6% after 2, 4 and 8 years following VL treatment, respectively [11].

In a study from 2013, 664 VL patients were treated with MF; 11 (2.5%) were suspected, and 5 (1.0%) were confirmed to have PKDL. No cases of PKDL were detected among VL patients treated with AMB.

A PKDL-prevention strategy should be adopted by the KAEP with advocacy aimed at clinicians for complete treatment of VL and sensitization of health workers for early recognition, referral, diagnosis and case management.

#### Discussion

It was mentioned that Nepal used three doses of liposomal amphotericin B (LAMB) for treatment of PKDL and that follow-up of these cases would be useful

### Observational cohort study in Bangladesh on the proportion of PKDL among the cured VL patients treated with monotherapy or combination (Presenter: Dinesh Mondal)

#### Overview

A follow-up study was carried out using an observational cohort of cured VL patients treated between August 2010 and April 2014, with one of the following regimens[12, 13]: (i) single intravenous infusion of LAMB (AMB), at a dose of 10 mg/kg (SDA); (ii) MF monotherapy at a dose of 50 mg BD (*bis in die*, or twice per day) for 28 days (MF); (iii) SSG monotherapy at a dose of 20 mg/kg intramuscularly (IM) for 28 days (SSG); (iv) paromomycin (PM) monotherapy at a dose of 11 mg/kg/day for 21 days IM (PMIM); (v) multiple-dose (MD) LAMB (AMB) at a dose of 5 mg/kg/day on days 1, 3 and 5 (MDAMB); (vi) combination of single intravenous infusion of LAMB at a dose of 5 mg/kg (AMB) followed by PMIM, 11 mg/kg/day for 10 days (PMIM+AMB arm); (vii) combination of single intravenous infusion of LAMB (AMB) at a dose of 5 mg/kg/day, followed by oral MF at a dose of 50 mg BD for 7 days (AMB+MF arm); (viii) combination of 11 mg/kg/day PMIM for 10 days and oral MF 50 mg BD for 7 days (PMIM+MF arm).

The aim of the study was to estimate the PKDL and visceral leishmaniasis relapse (VLR) rate following the above treatment regimens for VL. While the SSG cohort was retrospective, all other patients were followed prospectively, and data were analyzed using the Cox proportional hazard model.

#### Incidence of PKDL

The trend of PKDL incidence development peaks at year 3 after VL treatment and varies according to VL regimen. Of 984 cured VL patients that were followed up, 121 developed PKDL. The average incidence of PKDL was 14% (range 9–23%) over a period of 4 years. The incidence was lowest in the SSG arm, followed by MDAMB, MF, PMIM+AMB, SDAMB, AMB+MF, PMIM and PMIM+MF. For SSG and MDAMB the PKDL rates were significantly lower than the other regimens [13].

#### Incidence of VLR

VLR was found to peak one year after VL treatment. Of 984 participants, 69 developed VLR with a median time of 1.05 years (interquartile range, 0.77–1.53). The MDAMB, PMIM+MF, and PMIM+AMB regimens all showed significantly lower rates of VLR than the other regimens [13].

#### Conclusions

The treatment regimen for VL is significantly associated with PKDL and VLR. As SSG is no longer acceptable as a first line of treatment, MDAMB and AMB+PMIM have the lowest PKDL and VLR incidence rate, of which MDAMB seems the preferred option. After finalization of the analysis, a recommendation needs to be formulated and submitted to the KAEP [13].

#### Discussion

It may be better to restrict AMB to treating VL, as we should not widely depend on one drug for other indications, as well. Other meeting attendees disagreed, as MDAMB and AMB+PM show the lowest PKDL rates. Extending follow-up to over 4 years would be useful for the two arms that showed increase of PKDL rates. There was an adjustment for length of follow-up for the SSG retrospective data; otherwise, this adjustment was done using Cox proportional hazard model.

### General discussion on VL follow-up studies and implications

A short treatment duration with a strong drug is likely to be better for an immune shift, perhaps as a result of the more rapid reduction in parasite load. A Th2 response follows a high antigen dose, while in the case of a low antigen dose, a Th1 response follows. Genetic immune expression may be different at certain points in time, which may also play a role. Currently, SDA is the best treatment for VL. Maybe it is time to shift to a combination of AMB and PM. MDAMB may be better at preventing PKDL than SDAMB, but there is not enough evidence to change the program. A longitudinal study of immune profiles after treatment of VL is needed. It should be noted that immune profiles in the skin are totally different from those in the blood. A follow-up period of 5 years after VL treatment is recommended. More discussion is needed to produce a recommendation, as well as more data on the role of PKDL in transmission.

## Session: Diagnosis

### PKDL diagnostic tests in the hospital and in the field (Presenter: Poonam Salotra)

#### Overview

In 2017, there were 2000 PKDL cases in India, and this number is increasing. There are constraints in diagnosis: cultures are often not positive, are prone to contamination, and are therefore hardly used. Microscopy is very specific but has low sensitivity. Serological tests are easy and highly sensitive but have limited specificity (due to previous VL). There is no previous history of VL in 10–20% of PKDL cases; in a retrospective study over 2 decades, 20% had no previous VL, 80% cases of PKDL occurred over 12 months after VL, and 26% were initially misdiagnosed at the Primary Health Care level.

The preferred diagnostic has changed over time, from parasitology through serology [direct antiglobulin test, rK39 Rapid Diagnostic Test (RDT)], urine antigen detection tests (KAtex), and, ultimately, to nucleic acid detection with PCR, qPCR, and loop-mediated isothermal amplification (LAMP). qPCR is the most sensitive and specific assay for diagnosis of both VL and PKDL; it is also useful for assessment of cure and is widely used. A higher parasite load is a risk factor for relapse [14].

In practice, clinical diagnosis is usually performed in combination with the K39 RDT, as confirmatory molecular tests are not usually feasible. In a referral hospital, clinical diagnosis, rK39 RDT, slit skin smear, histopathology, and molecular tests are done. The rK39 RDT can also be done on a slit skin smear with equal performance.

In the field, no reliable tests are available. Potential options include isothermal amplification assays such as LAMP, for which no post-PCR steps are needed, as only a color change is detected, it is cost-effective and detects current infection [15]. LAMP has been validated at Benares Hindu University (BHU), Muzaffarpur, and the Rajendra Memorial Research Institute, Patna, and showed high sensitivity and specificity in both centers comparable with qPCR. Currently this test is being developed in kit form for use in the field.

#### Discussion

qPCR is the gold standard for diagnosis of PKDL, while LAMP is almost as good.

How long PCR remains positive after treatment of PKDL has not yet been examined.

### Development and evaluation of novel diagnostic tests for VL and PKDL (Presenter: Dinesh Mondal)

#### Overview

The gold standard for diagnosis of PKDL is currently microscopy and qPCR; the latter is best, as experience with microscopic examination of skin specimens shows that it is of low sensitivity.

A study was performed to determine whether LAMP on slit skin smears and whole blood and the urine Ag detection test could accurately diagnose PKDL. In this study, DNA was extracted from skin punch biopsies or whole blood by the QIAamp DNA Mini Kit® (Qiagen, Hilden, Germany) or the boil-and-spin method. The Urine Leishmania Antigen ELISA (enzyme-linked immunosorbent assay) was used to detect leishmanial antigen in urine. Results showed LAMP on skin-punch biopsy had 100% sensitivity using the Qiagen kit and 71% using the boil-and-spin DNA extraction method. LAMP on whole blood had a sensitivity of less than 10% in both methods. The ELISA test had sensitivity of 46%, and all tests showed 100% specificity. It was concluded that LAMP on a skin punch biopsy is field friendly and less expensive than qPCR and may be recommended as an alternative.

#### Discussion

Many PKDL patients do not present to specialized clinics, which could be a limitation. For the moment, this can be rolled out to the subdistrict level but not to the periphery.

### Experience of suitcase laboratory and future study

#### Presenter 1: Ahmed Abd El Wahed

A test kit is needed that is stable at temperatures of over 30 °C for field use. A mobile solar-powered suitcase laboratory was designed with a solar panel and a power pack. There is one suitcase for extraction and one for detection. Rapid extraction is needed to avoid errors due to the ambient heat. SpeedXtract^®^ (Qiagen, Hilden, Germany) is used because it is stable in the field for 3 months at high temperatures. It is faster than the Qiagen-DNeasy Plant Mini kit (15 min *vs* overnight, respectively). Even in case of a very low number of parasites, a positive signal can be obtained within 20 min. This is faster than qPCR. It not only gives a positive or negative signal but also differentiates between acute VL and VL that is in the initial treatment phase. Even a single parasite can be detected [16].

#### Presenter 2: Abhijit Sharma: future studies

A multicountry (Bangladesh, India, Nepal, Sri Lanka) single-blinded phase 2 study for rapid diagnostics is planned to determine the diagnostic accuracy of recombinase polymerase amplification assay *L. donovani* and qPCR for VL, PKDL, and cutaneous leishmaniasis (CL) in 1200 archived samples.

#### Discussion

Given that for every VL case there are nine asymptomatic infections, no treatment should be given to asymptomatic individuals who are PCR-positive. PCR may be useful in follow-up.

In this study there is no data on malaria and fungal infections of the skin, which might be a source of false positives. Assessing this would increase the cost. It would be best to use the current practice of clinical diagnosis and combine it with PCR.

### Experience of PKDL diagnostic algorithm in the field and active case finding (Presenter: Kingsuk Misra)

#### Overview

With trends of VL cases decreasing and PKDL cases increasing in India, West Bengal has initiated active case finding (ACF). As this was not very effective in detecting PKDL cases, a house-to-house survey search was initiated, with nodal camps for diagnosis. The World Health Organization (WHO) diagnostic algorithm was used under field conditions. Based on caseloads reported over the last two years, 481 villages were chosen with an estimated total population of 1.3 million. Every household was visited and increasing numbers of PKDL were found; there were 1067 suspected cases of PKDL, of whom 404 were probable PKDL cases. In Jharkhand, there were more cases than in Bihar; only four districts are endemic, encompassing 33 blocks (subdistricts). This form of ACF is very costly; the cost per village screened was GBP 1100–1900, and per VL + PKDL diagnosis this varied between GBP 1150–1500. In Jharkand this is done through the local health system. A limitation of the study was the likelihood of overdiagnosis, as diagnosis was done based on clinical examination, with rK39 RDT and a previous history of VL.

The key recommendation was that the WHO guidelines for diagnosis need updating. Furthermore, all probable cases need to be treated without waiting for a confirmatory test such as PCR on a slit skin smear. In addition, since MF is the first-line treatment for PKDL, a pregnancy test should be offered as well as counseling on contraceptive methods.

#### Discussion

In a study in India, 90% of all the probable PKDL cases are qPCR positive, according to the WHO algorithm; there are very few false-positive cases. Other experience showed that over 20% of cases initially diagnosed at health centers were misdiagnosed. This approach is costly. It might not be realistic for national programmes to continue with the door-to-door approach; however, it is important for elimination. An index case-based approach might be more realistic (i.e. only going to areas reporting VL cases). There are no data on alternative diagnoses, such as malaria or fungal diseases.

## Session: Immunology and vaccines

### Overview of basic immunology and vaccine targets in VL and PKDL (Presenter: Farrokh Modabber)

#### Overview

Leishmanization is the oldest and best form of immunization so far. Inoculation with live virulent *L.* *major* parasites induces a lesion that fades after 6 months. It was abandoned since inoculation did not induce a lesion in everyone. As the vaccine contains live parasites, it is not stable and thus not practical. The immune response is mainly Th1 with induction of cluster of differentiation 4 (CD4) cells after inoculation, with live parasites and CD8 if dead parasites are used.

The first-generation vaccines were from the New World. The Mayrink vaccine (Brazil) consisted of several leishmania strains; it was safe but not efficacious in prevention of CL. It was registered as a therapeutic vaccine (with low-dose antimony) in CL. Through the involvement of WHO’s Special Programme for Research and Training in Tropical Diseases (TDR), a single-strain vaccine was produced and tested in Colombia; it was safe but not efficacious as a prophylactic vaccine [17]. Later studies in Venezuela, by Convit, successfully used the autoclaved *L. mexicana* vaccine bacille Calmette-Guerin (BCG) as an immune enhancer for immunotherapy in CL [18].

In the Old World, the first-generation vaccines used killed *L. major* parasites. They aimed at producing leishmanin skin test (LST) conversion as a marker for protection. Initially autoclaved *L. major* vaccine was used with BCG as an immune enhancer. It was tested by WHO/TDR in both zoonotic and anthroponotic cutaneous leishmaniasis and found to be safe but not very efficacious. [19, 20]. In another TDR study, alum was added, which induced a strong immune response as measured by a strong LST conversion and high IFN-γ levels [21].

A prophylactic phase 2b proof-of-concept study was done in Sudan to prevent VL. In that study, 554 patients were recruited and randomized for receiving a single injection of the vaccine with BCG or BCG alone and then followed for 2 years. There were no cases of VL in the vaccinated group and four proven cases in the controls [22].

The same vaccine was used in the treatment of persistent PKDL cases which had been symptomatic for 6 months. It was found to be efficacious with LST conversion and increased IFN-γ/IL-10 production; CD8 cells predominated the immune response [23].

It is regrettable that the vaccine was not pursued. There were a few roadblocks: it was manufactured in Iran, standardization was difficult, and it requires fetal calf serum. It was, however, inexpensive, stable when autoclaved, and easy to produce. Newly defined vaccines were on the horizon.

The second-generation vaccines were based on trivalent antigens (thiol-specific antioxidant, *L. major* stress-inducible protein 1, and *Leishmania* spp. elongation and initiation factor) with additional antigens. The vaccine was produced by the Infectious Disease Research Institute, Seattle, USA (developed by S. Reed and R. Coler) [24, 25]. It aimed to stimulate the toll-like receptor 4. It was safe and immunogenic; antibody production was high. It showed some activity for CL but failed in the treatment of persistent PKDL in Sudan.

The ChAd63-KH vaccine (Paul Kaye, York, UK) is a third-generation vaccine. It was tested in Sudan and targeted initially for PKDL. It is safe, with around 100% IFN-γ production [26].

The LEISHDNAVAX vaccine uses a linear vector with two loops and a Th1 peptide, with or without a synthetic adjuvant. It is under development.

The fourth-generation vaccine should focus on live parasites as this is the most efficacious. One new development is the modification of virulence genes, with the parasites living long enough to induce the immune response before dying.

Another development is a live attenuated [27] *L. donovani* parasite vaccine with the centrin gene deleted (H. Nakhasi, US Food and Drug Administration).

In addition, there is an immunomodulator (CpG oligodeoxynucleotide) that only stimulates plasmacytoid dendritic cells; it induces maturation of monocytes into mature dendritic cells and is a strong Th1 adjuvant. It is under development by DND*i* in collaboration with the US Food and Drug Administration. (www.dndi.org)

In conclusion, nonprofit organizations with public-private partnership in endemic countries must be involved, with a devoted Pharma partner and a devoted vaccine organization.

#### Discussion

In order to measure protection in populations already exposed, it is possible to infect with knock-out parasites and give a live challenge, as has been done in mice [28]. We need better markers to identify who has been exposed. While it is the case that when administering BCG in controls there is some response, it is better not used as a stimulus in controls. Perhaps those who do and who do not respond might be distinguished genetically.

### Safety and immunogenicity of a new *Leishmania* vaccine candidate ChAd63-KH (*Leish2a*) (Presenter: Ahmed Musa)

#### Overview

Previous vaccine efforts have targeted CD4 cells by employing crude antigen mixtures, autoclaved parasites, or defined antigens. The ChAd6-KH vaccine targets CD8 cells rather than CD4 cells, as CD8 T cells are important for protection against leishmaniasis. In both asymptomatic patients and in treated VL patients, the frequency of activated CD8 cells is substantially increased. The ChAd63-KH vaccine uses a defective simian adenovirus that expresses a novel synthetic gene (KH) encoding two *Leishmania* proteins, KMP-11 and HASPB. It was developed by Paul Kaye of the University of York, UK [26]. Studies so far have shown that it has a good safety profile, is well tolerated, and induces a good immune response. It is currently being investigated in a phase 2a dose-escalating study in Sudan at the Professor El-Hassan Center in Dooka, Sudan. The low dose was well tolerated; of eight patients enrolled, one did not need medication and all others were treated with AMB. Of the eight patients enrolled for the high dose, three patients did not need further treatment and five showed improvement, with increased γ- interferon levels. Side effects were mainly grade 1 and 2: local redness, swelling and pain, transient lymphopenia, and headaches, similar to other adenoviral-vectored vaccines. If successful, a phase 2b study will be done with 90 participants (vaccine *vs* placebo), with follow-up for 39 days.

The ultimate targets for therapeutic vaccination are the prevention of transmission from asymptomatic patients, prevention of development of clinical VL disease, and prevention of PKDL and its potential role in transmission.

#### Discussion

The current status is that the lower dose was evaluated already, and green light was given for 2nd cohort. Clinical results from the higher dose were not presented here but from 8 patients, 3 on the higher dose did not need medication; 5 improved but still were treated with medication whereas with the lower dose, 1 did not need medication; all the others were treated with AmBisome.

### Targeted strategies for the prevention of leishmaniasis (Presenter: Malcolm Duthie)

#### Overview

The goal is to develop a CD4-targeting vaccine that can also be used as a prophylaxis. The LEISH-F3+GLA-SE vaccine induces an immune response entirely through the CD4 pathway and has been found to be safe and efficacious in humans [29]. The antigens are conserved along the spectrum of leishmaniasis. Recently another protein was added, truncated cysteine protease b. *Leishmania donovani* responds very well to this. The choice of adjuvant matters: GLA-SE substitutes give an inferior response. The vaccine protects mice against needle challenge with *L. donovani* or *L. infantum*; it protects hamsters against challenges with *L. donovani* by *L. longipalpis*. Future aims are to integrate sand fly salivary antigens and to do post-exposure assessments and clinical trial dose-escalating studies in endemic areas.

#### Discussion

There is a need to focus on an appropriate animal model (i.e. the hamster model is much better). The need for a live vaccine was repeated. This vaccine has potential in dogs, as T-cell exhaustion occurs in infected dogs, who respond to vaccination, while healthy controls do not.

## Session: Parasitology

### Parasite kinetics in PKDL (Presenter: Mitali Chatterjee)

#### Overview

Studies on PKDL were reported from West Bengal. Passive case finding surveillance was done since 2003, and 100 PKDL patients were recorded; ACF started in 2015 with 198 patients recorded. In contrast to passive case finding, where a male preponderance is found, there is no gender bias in ACF. Due to ACF, the patient delay is reduced from 3 to 2 years. Peak PKDL incidence is 3 years after VL treatment. In nine of ten patients, clinical diagnosis is confirmed by PCR. Baseline data on parasite burden using PCR showed a parasite load of 5229 µg gDNA (range 896–50,898) for PKDL in the skin as compared to 12,851 µg gDNA (range 2276–29,317) for VL in whole blood. Macular PKDL lesions had fewer parasites as compared to polymorphic lesions. In a study in which patients are randomly recruited and treated with MF (*n* = 79) and AMB (*n* = 96), 12 and 33 patients with macular lesions who were treated with MF and LAMB, respectively, were biopsied; for those with polymorphic lesions, 23 and 35 patients treated with MF and LAMB were biopsied, respectively. Those treated with MF cleared the lesions better, with clearance of parasites demonstrated by qPCR in the biopsies, while those treated with AMB had a less favorable clinical response with persistent parasites. This same pattern was found 6 months after treatment; and in the LAMB group, the parasite load increased again at that time point [30].

#### Discussion

Irrespective of treatment length, all patients were evaluated 6 months after start of treatment. LAMB may not be as effective as MF because of the pharmacokinetics of the skin and because of their different immunomodulatory properties. In addition, MF was given for a longer duration (3 months) while AMB was only given for 3 weeks. The maximum drop in parasite load was in the first 4 weeks; between weeks 4 and 8 the drop was only 10%. Macular lesions are a special category; the parasite load is low and hard to quantify before and after treatment. It is important to know when lesions cease to be infective. Macular lesions develop over time into polymorphic lesions, but the host immune response is also an important factor. Another factor is the different immune response in skin and visceral organs; transient expression of genes may occur. There is little information about the genetic profile of PKDL as opposed to VL.

### Issues in leishmanial strains in PKDL/VL, including resistance (Presenter: Poonam Salotra)

#### Overview

Unresolved issues in PKDL include: previous treatment of VL as a risk factor since 10–20% of patients do not have previous VL; whether PKDL occurs because of a reinfection with *Leishmania* parasites or due to the persistence of parasites; the role of duration of treatment; factors that determine the drive of parasites from the visceral organs to the skin and the role of PKDL in the spread of resistant/tolerant strains.

Using arbitrarily primed PCR, all strains from India show identical amplification [31]. A series of 178 strains from PKDL, susceptible or resistant to antimony, were identical in microsatellite typing using 11 markers [32]. Genetic studies on polymorphism showed minor genetic differences in strains from VL or PKDL from India and Nepal [31, 33, 34]. Differences in gene expression in strains of VL and PKDL occur in 2% of genes of PKDL strains, but some proteins are upregulated such as Gp63 and PSA [35]. These may alter the parasite and promote accumulation in the skin.

The treatment duration for PKDL (with SSG, MF, amphotericin B, or LAMB) may be 3 to 4 times longer than for VL. SSG-resistant PKDL isolates show considerably lower susceptibility to SSG compared to SSG-resistant VL strains, and these may contribute to increased SSG resistance in the community *via* anthroponotic transmission [36]. PKDL isolates show lower susceptibility to MF pretreatment compared to pretreatment of VL, while this is not the case for PM or amphotericin B [37]. It has been demonstrated that relapse after treatment of PKDL with MF reduced susceptibility *in vitro* as well as clinically, resulting in a declining efficacy of MF for PKDL treatment [38]. In a recent study, factors related to MF resistance were similar in VL and PKDL isolates as evidenced by increased gene expression of redox homeostasis, metabolic pathways, and transporters. Further studies include investigation of PKDL strains by whole genome sequencing and comparison of PKDL strains using genomic and proteomic approaches.

## Session: Treatment

### Treatment of PKDL in Asia and ongoing studies (Presenter: Shyam Sundar)

#### Antimony

In the 1990s, antimony (20 mg/kg, max 850 ml IM) was used; the response started within 20 days in 72% of the patients and within 40 days in all patients. All nodules and papules disappeared within 120 days, and the macules disappeared within 200 days. It was felt that it was not ethical to expose patients who are not ill to SSG at such high doses for a long duration [39].

#### Amphotericin B

In 1997 amphotericin B was used at a dose of 1 mg/kg/day on days 1 through 40 and 61 through 80 or with SSG 20 mg/kg/day with a 20-day drug-free interval; all patients were cured by amphotericin B with no relapses after 1-year follow-up, while this figure was only 63% for those treated with SSG [40]. Further studies at Rajendra Memorial Research Institute, Patna, India, with lower doses (0.5 mg rather than 1 mg and for 15 days rather than 20 days) showed similar cure rates but, surprisingly, more serious side effects [41].

#### Miltefosine

In various studies, miltefosine (MF) showed a 93–96% cure rate after 12 weeks, while this was only 81% after 8 weeks.[42–44] One study showed better results after 16 weeks over 12 weeks of MF [45]. Loss to follow-up was an issue in most studies. AMB in a high total dose of 30 mg/kg given in 3 weeks showed safety issues with rhabdomyolysis induced by hypokalemia in Bangladesh [46]. In a later study with a 15 mg/kg total dose, the cure rate was 90% with no adverse events [47].

There is an ongoing study in India and Bangladesh with AMB in monotherapy and in combination with MF; skin and plasma concentrations are being assessed.

Assessment of relapse is difficult in macular lesions; in nodular lesions, parasites reappear with clinical symptoms. Serology is not useful.

The declining efficacy of MF was demonstrated in a study by Ramesh et al. [38]. Cure rates for MF given at a dose of 50 mg BD for 3 months were 89.5% with 10.5% relapses and 68.8% with 31.3% relapses for 50 mg TDS for 2 months [38].

#### Paromomycin

In 31 patients (aged 10 years old or older) paromomycin (PM) was given in a dose of 11 mg/kg/day IM for 45 days; follow-up was for 1 year. Of those, 37.5% were cured while 62.5% showed improvement [48].

#### Conclusions

Treatment of PKDL is long and arduous. Antimony and amphotericin B regimens are impractical, with a high attrition rate. Oral MF is somewhat better but also has a long treatment duration, and its efficacy appears to be declining. Short-course LAMB shows promise. Better, preferably oral, drugs are needed.

#### Discussion

The reason why the response is worse for macular lesions than polymorphic lesions is poorly understood; only recently have macular cases in India been increasingly identified. Pharmacokinetic studies in the skin are needed.

Compliance to MF is poor because this treatment is associated with gastrointestinal side effects (nausea, vomiting), leading to interruption of treatment. This is particularly the case for VL (40%) but is less common for PKDL. Antiemetics may be considered for increased compliance.

The PKDL load might be stabilizing because expensive intensive case finding is happening less. It may also be that VL occurs in pockets, and door-to-door screening is needed.

Retinal ulcers that may proceed to blindness have been reported; they occur typically in males after 5 to 6 weeks of treatment.

### Treatment in Africa and ongoing studies (Presenter: Brima Younis)

#### Overview

In Sudan up to 62% of patients develop PKDL; in Ethiopia there are higher PKDL rates in patients with HIV coinfection. More effective therapy may produce lower PKDL rates: with SSG monotherapy 12% of patients develop PKDL, 9% with PM monotherapy, and 6% for SSG and PM combination therapy [49]. In Sudan, up to 85% heal spontaneously within 1 year [50]. Only those with grade 3 or grade 2 PKDL with severe disease are treated, or those with eye involvement, disfiguring disease, or oral lesions (children). The regimen is SSG 20 mg/kg for 30–60 days or SSG 20 mg/kg + PM 11 mg base/kg for 17 days until there is improvement only, rather than until all lesions have cleared. Follow-up is 12 months until all lesions have cleared. A limited study with SSG 20 mg/kg and PM 11 mg base/kg BD, both for 15 days, was done with good results (over 90% efficacy) but with moderate to severe pain at the injection site in most patients [51].

AMB is used as a second-line treatment. There is an ongoing study sponsored by DND*i* on PM and MF for 14 days, followed by another 28 days of MF monotherapy, *versus* short-course AMB (3 days) and MF for 28 days.

### PKDL treated with ambulatory short course AMB (Presenter: Margriet den Boer)

#### Overview

Médecins Sans Frontières aims to deliver free care for PKDL patients, including ACF with diagnosis and treatment. In a study in Bangladesh in 1918 patients, PKDL occurred in 0.1%, 2.7%, 8.9% and 10.7% of patients within 6, 12, 24 and 36 months, respectively. In another study, in 275 patients on the interval between VL and the occurrence of PKDL, PKDL occurred in 0.4%, 51.6%, 30.5%, 10.9% and 6.5% of patients, within 1 year, 1 to 4 years, 5 to 9 years, 10 to 14 years, and over 14 years, respectively. In clinically diagnosed PKDL patients in Bangladesh and India, with standardized assessment using photography, high-dose AMB 30 mg total dose (6 × 5 mg/kg over 2 weeks) showed approximately 80% complete resolution of lesions. Of over 1400 of patients monitored, rhabdomyolysis was found in 6 patients (5 of these young females), thought to be induced by hypokalemia; this complication is poorly understood [47].

In a new regimen using 15 mg/kg total dose (5 × 3 mg/kg over 3 weeks), no hypokalemia was found. The efficacy was the same as for the 30 mg/kg regimen with complete resolution of all nodular and popular lesions and major or complete repigmentation of macular lesions in 78.0% of patients. In 5.2%, new lesions developed. Limitations of the study included an unknown rate of self-healing, the lack of a test of cure, and an arbitrarily chosen 12-month follow-up [47].

It remains to be seen whether the doses given are still too high and whether shorter treatments should be considered. Combination treatment with SDA may be an option; in females of reproductive age, monotherapy with AMB may be considered.

#### Discussion

Potassium supplementation was only given if the pretreatment level was low. The selection of the lower-dose regimen was to avoid high doses of AMB. There was no difference in the duration of lesions in the two studies. Whereas for malaria, for example, a cure rate of under 90% is not good enough as the aim is to prevent death, for PKDL this may be acceptable as the aim is to interrupt transmission, and this is not a fatal disease.

### Treatment of severe PKDL in India (Presenter: V. Ramesh)

#### Overview

In severe PKDL, MF or AMB seem the best choices; the duration of treatment is guided by the clinical response. Any type of PKDL can relapse, during which the clinical type may change. Both mild and severe cases can relapse. MF is good to treat severe, genital lesions, but prolonged treatment may be needed. AMB is also preferred in relapses because the parasite load may be high.

#### Discussion

The decision about when to stop treatment depends on the clinical and parasitological response.

#### General discussion on treatment

While some advocate for monotherapy, others prefer combination therapy, such as MF and AMB for 1 month, due to efficacy and prevention of resistance as well as options for shorter treatment duration. The availability and safety profile obviously play a role; MF cannot be given to women of child-bearing age, and access is limited. It may be that no single approach is appropriate. The ongoing PK studies may suggest a tailored approach. A suggestion was made to harmonize the outcomes of clinical trials in PKDL.

MF is not distributed through WHO as there is no donation programme; the responsibility is at the governmental level. MF is produced by a single manufacturer; generic MF needs quality assurance. AMB is not provided for PKDL in the current donation programme, only for VL. A short-course, safe, and oral treatment for PKDL would be preferable. It is not known how efficacious treatment of PKDL needs to be to interrupt transmission.

A test of cure is needed; qPCR is ideal and needs to be developed for field use. Currently clinical assessment of cure is done at the field level; an alternative would be to send samples for qPCR to a reference laboratory. Standardized criteria for cure would be useful both in the field and at the central level.

## Session: Xenodiagnosis and modelling

### Xenodiagnoses of VL, PKDL, and asymptomatic subjects using phlebotomine sand flies (Presenter: Om Prakash Singh)

#### Overview

In this ongoing study, only direct xenodiagnosis is used. Quality issues include identifying with certainty that only *P. argentipes* is in the colony and that the colony is virus-free. Only PKDL patients who are PCR-positive in skin and blood were selected. Nodular PKDL is more infectious than macular PKDL. Asymptomatic patients are defined as having a high titer in a direct antiglobulin test or ELISA; so far, no sand flies have been found to be infected from this group, nor from cured VL subjects.

#### Discussion

PKDL was diagnosed through positive skin slit smear by microscopy or PCR in whole blood. Sand flies feed in early hours of the morning, with the highest density at midnight and the highest number fed at 4:00 h. Although after 5 days there would be more infections, the authors dissected after 60 hours because if too much time passes the flies will die; this was a study of infection in sand flies and not infectiousness.

### Infectivity study in Bangladesh (Presenter: Jorge Alvar)

#### Overview

A combination of direct and indirect xenodiagnosis was carried out on 47 PKDL and 10 VL patients. The 47 PKDL patients were all confirmed by PCR; of these, 68% were also positive by microscopy. Macular lesions were found in 26 patients, and 21 had nodular lesions. Xenodiagnosis was done by inserting a hand or another part of the body inside a cage with sand flies. The parasite load was quantified by qPCR. No healthy skin was tested. Indirect xenodiagnosis was used for VL. In PKDL patients, 57% were infectious; in nodular cases, 18 (67%); and in macular cases, 9 (33%). The parasite load was 10-fold higher in nodular lesions than in macular lesions. In VL patients, 10 out of 15 were infective [52].

#### Discussion

The question of whether low numbers of PKDL will contribute much to transmission in the presence of high numbers of VL may be addressed by modelling. In the recent outbreak in Spain, HIV/VL coinfected patients were shown to be highly infective.

Sand flies feed on all parts of the body. It is difficult to obtain ethical clearance for feeding experiments on all parts of the body. Since one colony feeds better than another, results are best pooled. Both PKDL lesions and healthy skin are infectious to sand flies; macular lesions may be less infectious than healthy skin, but data are scanty. There is a need to define which category of patients contributes most, such as HIV coinfected patients.

The xenodiagnosis studies using reared sand flies may differ from those using wild sand flies that are more competent as a vector.

### Modelling transmission in Bangladesh (Presenter: L. Chapman)

#### Overview

The incidence of VL was already declining before the start of the elimination initiative; this is most likely the result of the epidemiological cycle. Studies in Bangladesh show that the PKDL peak follows the VL peak with an offset of 2 years. In the model presented it was shown that whether asymptomatic patients are infective or non-infective, PKDL still drives the transmission to a large extent. A limitation of the model is that spatial heterogeneity is not taken into account; the risk of transmission decreases by 50% within 100 m when compared to those living in the same household. Seasonality is another factor that should be considered in the model. A stochastic rather than a deterministic model would be preferable, as the number of PKDL cases is so small.

#### Discussion

Reporting of VL and PKDL may have improved when better treatments became available, impacting peaks in the epidemic curve, and this should be included in the model. It may be good to include indoor as well as outdoor transmission. It is not known at what VL/PKDL ratio PKDL is not significant to disease transmission, but this is an important issue to consider.

### The role of PKDL when nearing elimination on the ISC (Presenter: Epke Le Rutte)

#### Overview

The model uses data from Bihar, India. In the attack phase, ACD and insecticide residual spraying (IRS) are applied. When nearing elimination, there will be a large susceptible population. Questions to be addressed include how long to continue the current approach if, after 10 years, ACD and IRS are stopped indefinitely.

Two models are used. In the model E_0_ it is assumed that 50% of PKDL cases are infective to sand flies as compared to 100% of VL cases. In this model, PKDL contributes greatly to transmission. In model E_1_ it is assumed that 1–2% of asymptomatic cases are also infective; in this case PKDL still contributes around 50%. If IRS and ACD are stopped, VL will increase again. The impact of a vaccine to prevent PKDL from developing decreases the incidence of PKDL, but only slowly if no other interventions are applied. If the vaccine is added at the end of the consolidation phase, it is enough to maintain the low incidence of VL [53].

### Discussion on the modelling presentations (summary)

The cost-effectiveness of various approaches should be addressed. The issue of life-long immunity after treatment of VL and PKDL still needs to be addressed in the models. Sources of infection post-elimination include PKDL, newborns, and immigrants. Different treatment regimens for VL and PKDL, the VL/PKDL ratio and nodular *vs* macular PKDL ratio, and indoor or outdoor transmission should also be considered for inclusion in the models. Given these findings in infectivity in the ISC, treating all cases in Sudan may be considered. However, most patients recover spontaneously, and those with PKDL of under 6 months’ duration respond well to treatment, probably because they are self-healing anyway. The infectivity of this group should be established before a decision is made. Perhaps only those who persist or have severe PKDL should be treated to interrupt transmission; these cases are treated currently anyway. In any case, safe and efficacious short-course drug regimens are needed, as now only SSG, SSG +PM, or AMB are available.

The PKDL caseload is estimated to be around 4000 patients. Active PKDL case finding is also essential in terms of cost-effectiveness for the elimination phase of the KAEP. A vaccine preventing PKDL after VL could be even more cost-effective in the future. In the absence of a vaccine, combination treatment of VL may also reduce the PKDL rate. Socioeconomic development in certain areas should also be considered as this may reduce recrudescence.

## Overall conclusions and summary of the meeting

The meeting brought together the majority of global PKDL experts, including clinicians, basic scientists and policymakers. After reviewing and extensively discussing all the available information, four priorities were identified:

(i) Understand the role of PKDL in transmission. Increasing evidence shows that PKDL can infect sand flies, and this is true for both macular and papular/nodular lesions, although the latter contributes most. There is a need to:
  standardize the methodology used (PCR, qPCR, etc.);
  harmonize protocols by listing the need for studies in asymptomatic, ex-VL, VL, HIV-VL, and ex-PKDL patients, including numbers needed in each region: Asia (India, Bangladesh) and Africa (Sudan); and
  continue refining epidemiological models to be used in decision-making, such as in the KAEP.
(ii) Further analyze the PKDL rates following VL treatment in both India and Bangladesh, to recommend an optimal regimen for VL with respect to prevention of PKDL. Clearly such a regimen will also be defined by cost, practical issues, resistance development, and other factors.
(iii) Interpret ongoing PKDL treatment studies in relation to parasite clearance induced by various drugs and the implications for relapse and cure, as well as the role of reservoirs.
(iv) Promote the need for a field-based diagnostic, preferably a molecular tool such as LAMP. In the meantime, the diagnostic algorithm as defined by WHO may be revised and adapted for hospital-based and field-based diagnosis. Similar efforts pertain to the need for a biomarker to assess the response to treatment.

## Supplementary information

**Additional file 1.** Consortium meeting agenda.

**Additional file 2.** Consortium meeting attendees.

## Data Availability

Not applicable.

## Abbreviations

ACD: active case detection
ACF: active case finding
AMB: AmBisome
BCG: bacille Calmette-Guerin
BD: *bis in die* (twice per day)
BHU: Benares Hindu University
CD: cluster of differentiation
CL: cutaneous leishmaniasis
ICDDR,B: International Centre for Diarrheal Diseases Research, Bangladesh
IFN-y: interferon-gamma
IL-10: interleukin-10
IM: intramuscularly
IRS: insecticide residual spraying
ISC: Indian subcontinent
KA: kala-azar
KAEP: Kala-Azar Elimination Programme
LAMB: liposomal amphotericin B
LAMP: loop-mediated isothermal amplification
LST: leishmanin skin test
MD: multiple-dose
MF: miltefosine
PCF: passive case finding
PCR: polymerase chain reaction
PKDL: post-kala-azar dermal leishmaniasis
PM: paromomycin
qPCR: quantitative polymerase chain reaction
RDT: rapid diagnostic test
SDA: single-dose AmBisome
SSG: sodium stibogluconate
TDR: Special Programme for Research and Training in Tropical Diseases
Th: T-helper
VL: visceral leishmaniasis
VLR: visceral leishmaniasis relapse
