# Supplementary material for: Report of the Fifth Post-Kala-Azar Dermal Leishmaniasis Consortium Meeting, Colombo, Sri Lanka, 14–16 May 2018
Source: Parasit Vectors. 2020 Mar 30;13:159. doi: 10.1186/s13071-020-04011-7 (PMC7106569; doi:10.1186/s13071-020-04011-7)
Supplement: Supplementary file 1 — Additional file 1. Consortium meeting agenda. [file 13071_2020_4011_MOESM1_ESM.docx]

**Additional file 1.** Consortium meeting agenda

Post**-**Kala**-**Azar Dermal Leishmaniasis (PKDL) Consortium Meeting

May 14**–**16, 2018, Colombo, Sri Lanka

## Agenda breakdown by day

Table S1. Day 1 agenda—May 14, 2018 (Monday).

| **Time** | **Session Name/Description** | **Participants/Presenters** | **Session Length** |
| --- | --- | --- | --- |
| 08:30–09:00 | Registration | All | 30 min |
| 09:00–10:10 | Inauguration | Chair: *Suman Rijal, Amresh Kumar* | 1 hour 10 min |
|  | Welcome & objectives of the meeting | Ed Zijlstra (on behalf of organizing committee) | 10 min |
|  | Opening remarks | Neeraj Jain (PATH) | 5 min |
|  | Relevance of PKDL in VL Elimination Program in South Asia | Saurabh Jain (WHO) | 10 min |
|  | Country perspective for PKDL control | India | 10 min |
|  |  | Bangladesh | 10 min |
|  |  | Nepal | 10 min |
|  | Keynote address | Director General of Health Services (Sri Lanka) | 5 min |
|  | Vote of thanks | Dean of Faculty (USJ) | 5 min |
|  | Vote of thanks | Satyabrata Routray (PATH) | 5 min |
| 10:10–10:40 | Tea/coffee break | All | 30 min |
| 10:40–12:35 | Epidemiology & disease burden and clinical presentation | Chair: *Be-Nazir Ahmed, Farrokh Modabber* | 1 hour 55 min |
|  | Epidemiology of PKDL in Asia and Africa | Ed Zijlstra | 30 min |
|  | PKDL observational study (4 years f/up) Bangladesh result | Amresh Kumar and Dinesh Mondal | 20 min |
|  | PKDL observational Study (24 months f/up) India result | Suman Rijal | 20 min |
|  | Discussion on  experience of PKDL in the field | All | 45 min |
| 12:35–13:35 | Lunch | All | 1 hour |
| 13:35–14:55 | Diagnosis | Chair: *Fabiana Alves, Mitali Chatterjee* | 1 hour 20 min |
|  | PKDL diagnostic tests in the hospital and in the field | Poonam Salotra | 20 min |
|  | Diagnostic tests currently under development | Dinesh Mondal/ Isra Cruz | 20 min |
|  | Experience of suitcase lab and future study | Ahmed Abd El Wahed and Abhijit Sharma | 20 min |
|  | Experience of PKDL diagnostic algorithm in the field and active case finding | Kingsuk Misra | 20 min |
| 14:55–15:25 | Tea/coffee break | All | 30 min |
| 15:25–16.25 | Immunology and vaccines | Chair: *Asrat Hailu, Poonam Salotra* | 1 hour |
|  | Overview of basic immunology and vaccine targets in VL and PKDL | Farrokh Modabber | 30 min |
|  | Ongoing vaccine study in Sudan | Ahmed Musa | 30 min |
| 16:25–17:10 | Future role of the PKDL Consortium and how we can sustain this | *Neeraj Jain, Ed Zijlstra, Jorge Alvar, Saurabh Jain, PK Sen* | 45 min |
| 17:10 | Adjourn for the day |  |  |
| 20:00–22:00 | Dinner | All / hosted by PATH | 2 hours |

*Abbreviations*: PKDL, post-kala-azar dermal leishmaniasis; USJ, University of Sri Jayewardenepra; VL, visceral leishmaniasis; f/up, follow-up.

Table S2. Day 2 agenda—May 15, 2018 (Tuesday).

| **Time** | **Session Name/Description** | **Participants/Presenters** | **Session Length** |
| --- | --- | --- | --- |
| 09:00–09:20 | Summary of Day 1  PKDL and KAEP in Asia | Suman Rijal | 20 min |
| 09:20–10:30 | Parasitology | Chair: *Ahmed Musa, Dinesh Mondal* | 1 hour 10 min |
|  | Parasite kinetics in PKDL | Mitali Chatterjee | 20 min |
|  | Issues in leishmanial strains in PKDL/VL including resistance | Poonam Salotra | 20 min |
|  | Discussion | All | 30 min |
| 10:30–11:00 | Tea/coffee Break | All | 30 min |
| 11:00–13:00 | Treatment | Chair: *Jorge Alvar, Saurabh Jain* | 2 hours |
|  | Treatment of PKDL in Asia and ongoing studies | Shyam Sundar | 25 min |
|  | Treatment of PKDL in Africa and ongoing studies | Brima Younis | 25 min |
|  | Recent clinical studies in PKDL: Médecins Sans Frontières experience | Margriet den Boer | 20 min |
|  | PKDL treatment of severe PKDL in India | V. Ramesh | 20 min |
|  | Panel discussion: Experience and challenges in the treatment in South Asia and Africa | Shyam Sundar/Be-Nazir Ahmed/Arpana Rijal/Margriet den Boer / V. Ramesh | 30 mins |
| 13:00–14:00 | Lunch | All | 1 hour |
| 14:00–16:00 | Xenodiagnosis and modelling | Chair: *Shyam Sundar, Ed Zijlstra* | 2 hours |
|  | Infectivity study in India | Om Prakash Singh/ Shyam Sundar | 20 min |
|  | Infectivity study in Bangladesh | Jorge Alvar, Dinesh Mondal | 20 min |
|  | Modelling leishmaniasis transmission in Bangladesh | Lloyd Chapman/ Graham Medley | 20 min |
|  | Progress in epidemiological modelling | Epke Le Rutte | 20 min |
|  | Discussion | All | 40 min |
| 16:00 | Adjourn for the day |  |  |

*Abbreviations*: KAEP, Kala-Azar Elimination Program; PKDL, post-kala-azar dermal leishmaniasis; VL, visceral leishmaniasis.

Table S3. Day 3 agenda—May 16, 2018 (Wednesday).

| **Time** | **Session Name/Description** | **Participants/Presenters** | **Session Length** |
| --- | --- | --- | --- |
| 09:00–10:00 | Group work | Discussion leader |  |
|  | - PKDL and elimination targets - Long- and short-term research needs - TPP for PKDL treatments - Advocacy and fund-raising | Suman Rijal  Dinesh Mondal, Ahmed Musa  Fabiana Alves  Amresh Kumar | 60 min |
| 10:00–10:30 | Tea/coffee break |  | 30 min |
| 10.30–12:15 | Final discussion and conclusions | Chair: *Ed Zijlstra, Suman Rijal* | 1 hour 45 min |
| 10:30–11:15 | Presentations, group work | All | 45 min |
| 11:15–11:45 | Rapporteurs report | All | 30 min |
| 11:45–12:15 | Recommendations | All | 30 min |
| 12:15–12:30 | Closing remarks | Ed Zijlstra & Satyabrata Routray | 15 min |
| 12:30 | Meeting adjourns |  |  |
| 12:30–13:30 | Lunch | All | 1 hour |

*Abbreviations*: PKDL: post-kala-azar dermal leishmaniasis; TPP: Target Product Profile.

## Organizers

Amresh Kumar

Dinesh Mondal

Suman Rijal

Abhijit Sharma

Ed Zijlstra

##

## Rapporteurs

Abhijit Sharma

Kristien Cloots

Margriet den Boer

Vijayashree Yellappa
