# Supplementary material for: Report of the Fifth Post-Kala-Azar Dermal Leishmaniasis Consortium Meeting, Colombo, Sri Lanka, 14–16 May 2018
Source: Parasit Vectors. 2020 Mar 30;13:159. doi: 10.1186/s13071-020-04011-7 (PMC7106569; doi:10.1186/s13071-020-04011-7)
Supplement: Supplementary file 2 — Additional file 2. Consortium meeting attendees. [file 13071_2020_4011_MOESM2_ESM.docx]

**Additional file 2.** Consortium meeting attendees

Table S4. List of attendees (with affiliations).

|  | **Name** | **Institutions/Organizations** |
| --- | --- | --- |
| 1 | Dr. Amresh Kumar | PATH, New Delhi, India |
| 2 | Dr. Abhijit Sharma | PATH, New Delhi, India |
| 3 | Mr. Rakesh Kumar Mishra | PATH, New Delhi, India |
| 4 | Dr. Satyabrata Routray | PATH, New Delhi, India |
| 5 | Prof. V. Ramesh | Vardhman Mahavir Medical College & Safdarjang Hospital, New Delhi, India |
| 6 | Prof. Shyam Sundar | Benares Hindu University, Varanasi, India |
| 7 | Dr. Mitali Chatterjee | Institute of Postgraduate Medical Education and Research, Kolkata, India |
| 8 | Prof. Poonam Salotra | National Institute of Pathology, New Delhi, India |
| 9 | Prof. Be-Nazir Ahmed | Directorate General Health Services, Dhaka, Bangladesh |
| 10 | Dr. Dinesh Mondal | ICDDR,B, Dhaka, Bangladesh |
| 11 | Dr. Rashidul Haque | ICDDR,B, Dhaka, Bangladesh |
| 12 | Dr. AFM Akhtar Hossain | National Kala-azar Elimination Program, Directorate General Health Services, Dhaka, Bangladesh |
| 13 | Dr. Bibek Kumar Lal | EDCD, Ministry of Health and Population, Kathmandu, Nepal |
| 14 | Dr. Surendra Uranw | BP Koirala Institute of Health Sciences, Kathmandu, Nepal |
| 15 | Dr. Arpana Sharma Rijal | BP Koirala Institute of Health Sciences, Kathmandu, Nepal |
| 16 | Prof. Ahmed Mudawi Musa | Institute of Endemic Diseases, Khartoum, Sudan |
| 17 | Dr. Brima Musa Younis Mohammed | Institute of Endemic Diseases, Khartoum, Sudan |
| 18 | Prof. Asrat Hailu | Addis Abeba University, Addis Abeba, Ethiopia |
| 19 | Dr. Ahmed Abd El Wahed | University of Göttingen, Göttingen, Germany |
| 20 | Dr. Vijayashree Yellappa | SPEAK India, New Delhi, India |
| 21 | Dr. Om Prakash | Benares Hindu University, Varanasi, India |
| 22 | Dr. Md. Sohel Shomik | ICDDR,B, Dhaka, Bangladesh |
| 23 | Dr. Subhash Lakhe | Communicable Disease Unit, World Health Organization (WHO), Nepal |
| 24 | Dr. Saurabh Jain | WHO, Geneva, Switzerland |
| 25 | Dr. Suman Rijal | Drugs for Neglected Diseases *initiative* (DND*i*), New Delhi, India |
| 26 | Dr. Margriet Leontine den Boer | Médecins sans Frontières, Operational Center Amsterdam, The Netherlands |
| 27 | Dr. S Sridhar, Care India | Care India, Delhi, India |
| 28 | Dr. Kingsuk Misra | KalaCORE India |
| 29 | Dr. Malcom Scott Duthie | Infectious Disease Research Institute, Seattle, United States |
| 30 | Dr. Farrokh Modabber | DND*i*, Geneva, Switzerland |
| 31 | Dr. Mary Mcfadden Cameron | London School of Hygiene and Tropical Medicine, London, UK |
| 32 | Dr. Lloyd Chapman | London School of Hygiene and Tropical Medicine, London, UK |
| 33 | Dr. Jorge Pablo Alvar | DND*i*, Geneva, Switzerland |
| 34 | Dr. Fabiana Alves | DND*i*, Geneva, Switzerland |
| 35 | Dr. Severine Monnerat | DND*i*, Geneva, Switzerland |
| 36 | Dr. Ed Zijlstra | DND*i*, Geneva, Switzerland |
| 37 | Epke Le Rutte | Erasmus MC, Rotterdam, The Netherlands |
| 38 | Dr. Sheeraj Raja | DND*i*, India office, New Delhi, India |
| 39 | Dr. Epco Hasker | Institute of Tropical Medicine, Antwerp, Belgium |
| 40 | Dr. Kristien Cloots | Institute of Tropical Medicine, Antwerp, Belgium |
| 41 | Dr. Shalindra Ranasinghe | University of Sri Jayewardenepra, Colombo, Sri Lanka |
| 42 | Prof. Sampath Amaratunga | University of Sri Jayewardenepra, Colombo, Sri Lanka |
| 43 | Dr. Anil Jasinghe | Directorate General Health Services, Colombo, Sri Lanka |
| 44 | Dr. Deepa Gamage | Ministry of Health, Colombo, Sri Lanka |
| 45 | Dr. Yamuna Siriwardana | Faculty of Medicine, University of Colombo, Sri Lanka |

*Abbreviation*: EDCD, Epidemiology and Disease Control Division; ICDDR,B, International Center for Diarrheal Diseases Research, Bangladesh; SPEAK Setting the Post-Elimination Agenda for Kala-azar in India
